# Dietary arginine affects the insulin signaling pathway, glucose metabolism and lipogenesis in juvenile blunt snout bream *Megalobrama amblycephala*

**DOI:** 10.1038/s41598-017-06104-3

**Published:** 2017-08-11

**Authors:** Hualiang Liang, Habte-Michael Habte-Tsion, Xianping Ge, Mingchun Ren, Jun Xie, Linghong Miao, Qunlan Zhou, Yan Lin, Wenjing Pan

**Affiliations:** 1 Wuxi Fisheries College, Nanjing Agricultural University, Wuxi, 214081 China; 2 Key Laboratory for Genetic Breeding of Aquatic Animals and Aquaculture Biology, Freshwater Fisheries Research Center (FFRC), Chinese Academy of Fishery Sciences (CAFS), Wuxi, 214081 China; 3 Division of Aquaculture, College of Agriculture, Food Science and Sustainable Systems (CAFSSS), Kentucky State University, 103 Athletic Road, Frankfort, KY 40601 USA

## Abstract

This study evaluated the mechanisms governing insulin resistance, glucose metabolism and lipogenesis in juvenile fish fed with graded levels of dietary arginine. The results showed that, compared with the control group (0.87%), 2.31% dietary arginine level resulted in the upregulation of the relative gene expression of IRS-1, PI3K and Akt in the insulin signaling pathway, while 2.70% dietary arginine level led to inhibition of these genes. 1.62% dietary arginine level upregulated glycolysis by increasing GK mRNA level; 2.70% dietary arginine level upregulated gluconeogenesis and resulted in high plasma glucose content by increasing PEPCK and G6P mRNA level. Furthermore, 2.70% dietary arginine level significantly lowered GLUT2 and increased PK mRNA levels. 1.62% dietary arginine level significantly upregulated ACC, FAS and G6PDH mRNA levels in the fat synthesis pathway and resulted in high plasma TG content. These results indicate that 1.62% dietary arginine level improves glycolysis and fatty acid synthesis in juvenile blunt snout bream. However, 2.70% dietary arginine level results in high plasma glucose, which could lead to negative feedback of insulin resistance, including inhibition of IRS-1 mRNA levels and activation of gluconeogenesis-related gene expression. This mechanism seems to be different from mammals at the molecular level.

## Introduction

Arginine is a multifaceted compound. Although it is abundant in the diet of most omnivorous and carnivorous fish species, it is still considered an essential amino acid as a natural component of dietary proteins for fish^1^. Additionally, L-arginine (L-Arg) is involved in protein metabolism^2^, the production of nitric oxide (NO) and the synthesis of creatine, L-ornithine, L-glutamate, polyamines and agmatine^3^. Insulin resistance is a state of decreased responsiveness of target tissues to normal circulating levels, resulting in glucose intolerance, obesity, dyslipidemia, all of which are symptoms of metabolic syndrome^3^. The IRSs/PI3K/Akt pathway plays an important role in insulin resistance^4,5^. Insulin receptor substrate (IRS) proteins act as messenger molecule-activated receptors to signaling, which are important steps of insulin action^6^. Phosphoinositide 3-kinase (PI3K) and protein kinase B (Akt), two major nodes downstream of insulin receptor substrate 1 (IRS-1), have been implicated in many of the metabolic actions of insulin^7^. Unlike mammals, the utilization of carbohydrates in fish is limited; feeding with high levels of carbohydrates results in persistent hyperglycemia and reduced growth performance^8^, which is similar to the insulin resistance phenomenon observed in mammals.

Previous studies found that dietary L-Arg supplementation affects energy metabolism, increases lipid oxidation and enhances the deposition of both lipids and proteins in muscle^9–13^. Recent studies revealed that L-Arg is also a potent secretagogue of the endocrine system, as it induces glucagon secretion from the pancreas and promotes growth hormone secretion from the anterior pituitary gland^14,15^, furthermore, L-Arg produces insulin^16^. There is growing evidence from mammals, including human studies, indicating that the physiological levels of L-Arg improve insulin sensitivity, which promotes the utilization of glucose^12,13^. The mechanism of improving insulin sensitivity has become a topic of focus in mammal-related research. In addition, L-Arg promotes the oxidation of long-chain fatty acids (LCFA) and decreases the synthesis of triacylglycerols *de novo*^10^. Nevertheless, there is little knowledge about the effects of arginine supplementation on the insulin signaling pathway and nutritional metabolism in fish.

The blunt snout bream, *Megalobrama amblycephala*, also known as the Wuchang bream, is a commercially important freshwater fish species in China with a long history of cultivation because of its excellent flesh quality, rapid growth and high larval survival rate^17,18^. It is also found in North America (northern Canada to southern Mexico), Africa and Eurasia^12^. The dietary arginine requirement of juvenile blunt snout bream has been determined^2,18^. In our previous study, we reported that the ribosomal protein S6 kinase 1 (S6K1) was overexpressed in response to high dietary arginine levels^2^; S6K1 overexpression might result in insulin resistance through a negative feedback mechanism^19^, thereby affecting glucose metabolism in juvenile blunt snout bream. This phenomena suggested that dietary arginine levels could affect the insulin signaling pathway. To the best of our knowledge, there is no information concerning the effects of dietary arginine supplementation on the insulin signaling pathway and lipid metabolism-related signaling molecules in fish. Therefore, the aim of this study was to investigate the effects of dietary arginine levels on the relative gene expression of the insulin signaling pathway, as well as glucose and lipid metabolism-related signaling molecules in juvenile blunt snout bream.

## Results

### Relative gene expression of the insulin signaling pathway in the liver

**Figure 1:**
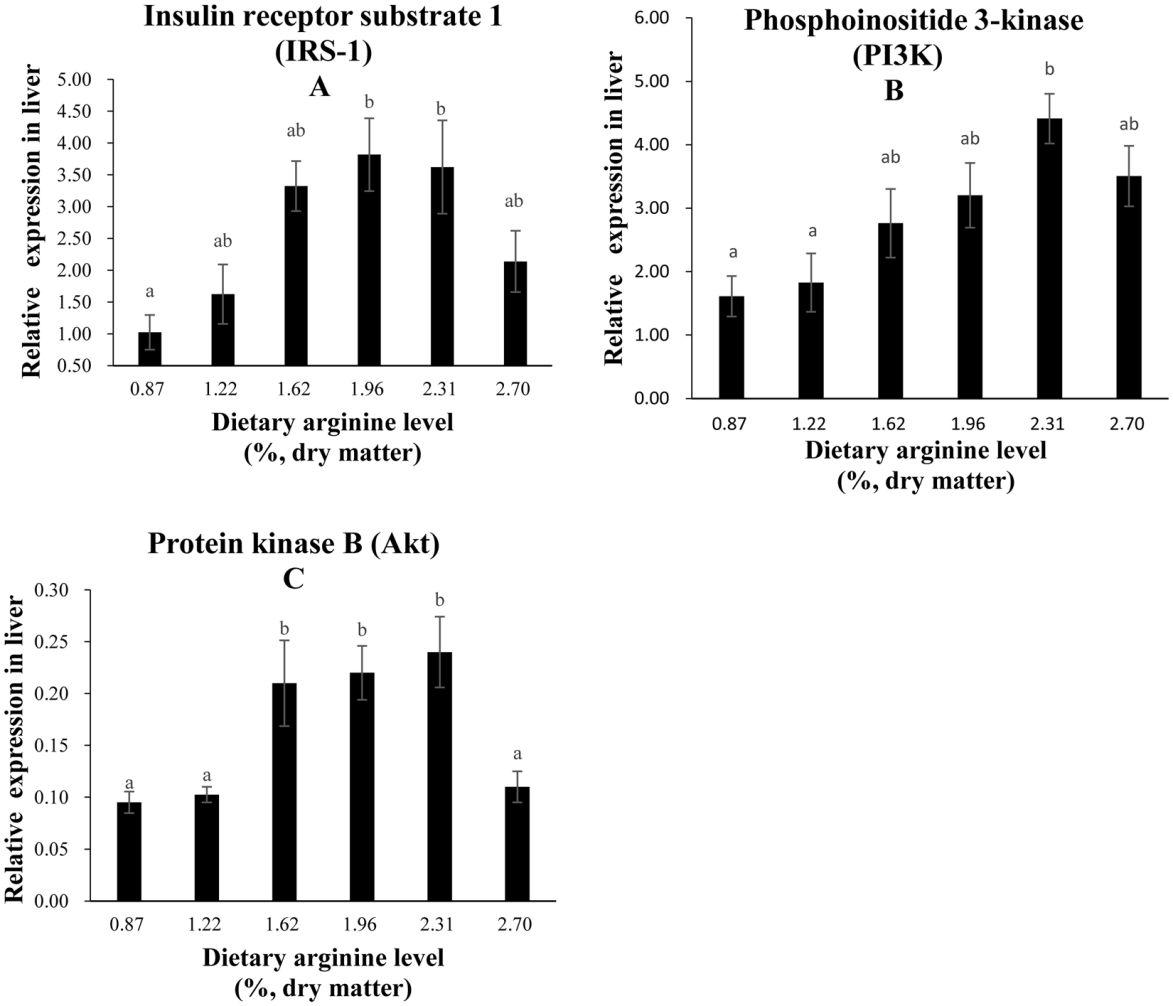
Relative expressions of insulin signaling pathway. Such as IRS-1 (**A**), PI3K (**B**) and Akt (**C**) genes in the liver of blunt snout bream fed diets with different arginine levels. Data are expressed as means with SEM. Value with different superscripts are significantly different (*P* < *0.05*).

Compared with the control group, the relative expression of IRS-1 in the groups fed with 1.96 and 2.31% dietary arginine were significantly upregulated and then showed a declining trend (Fig. 1A). Dietary arginine levels of 2.31% significantly increased the relative expression of PI3K (Fig. 1B). The relative expression of Akt in the groups fed with dietary arginine levels of 1.62, 1.96 and 2.31% were significantly upregulated and thereafter showed a decreasing trend (Fig. 1C).

### Glycometabolism in the liver

**Figure 2:**
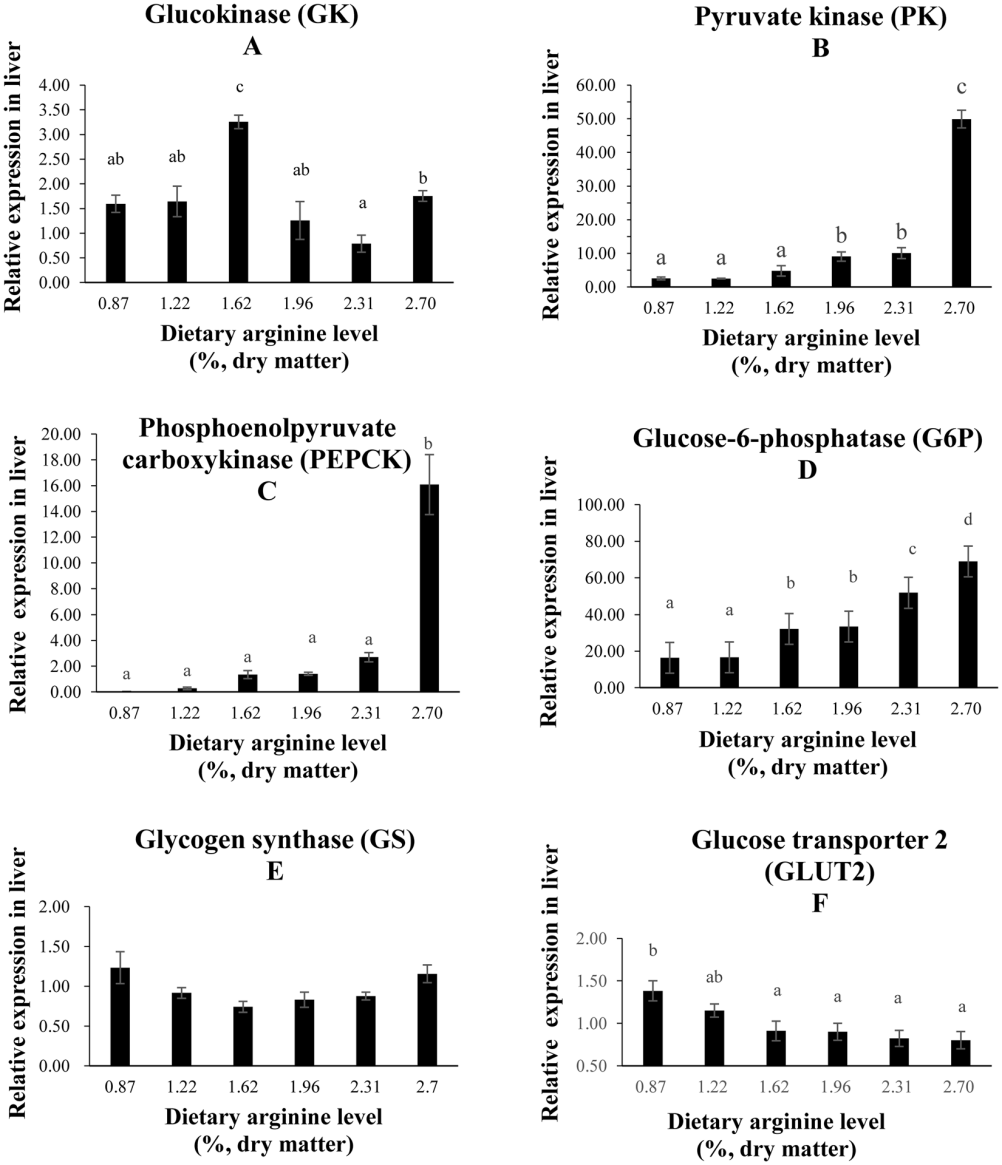
Relative expressions of glucose metabolism signaling pathway. Such as GK (**A**), PK (**B**), PEPCK (**C**), G6P (**D**), GS (**E**), and GLUT 2 (**F**) genes in the liver of blunt snout bream fed diets with different arginine levels. Data are expressed as means with SEM. Value with different superscripts are significantly different (*P* < *0.05*).

**Figure 3:**
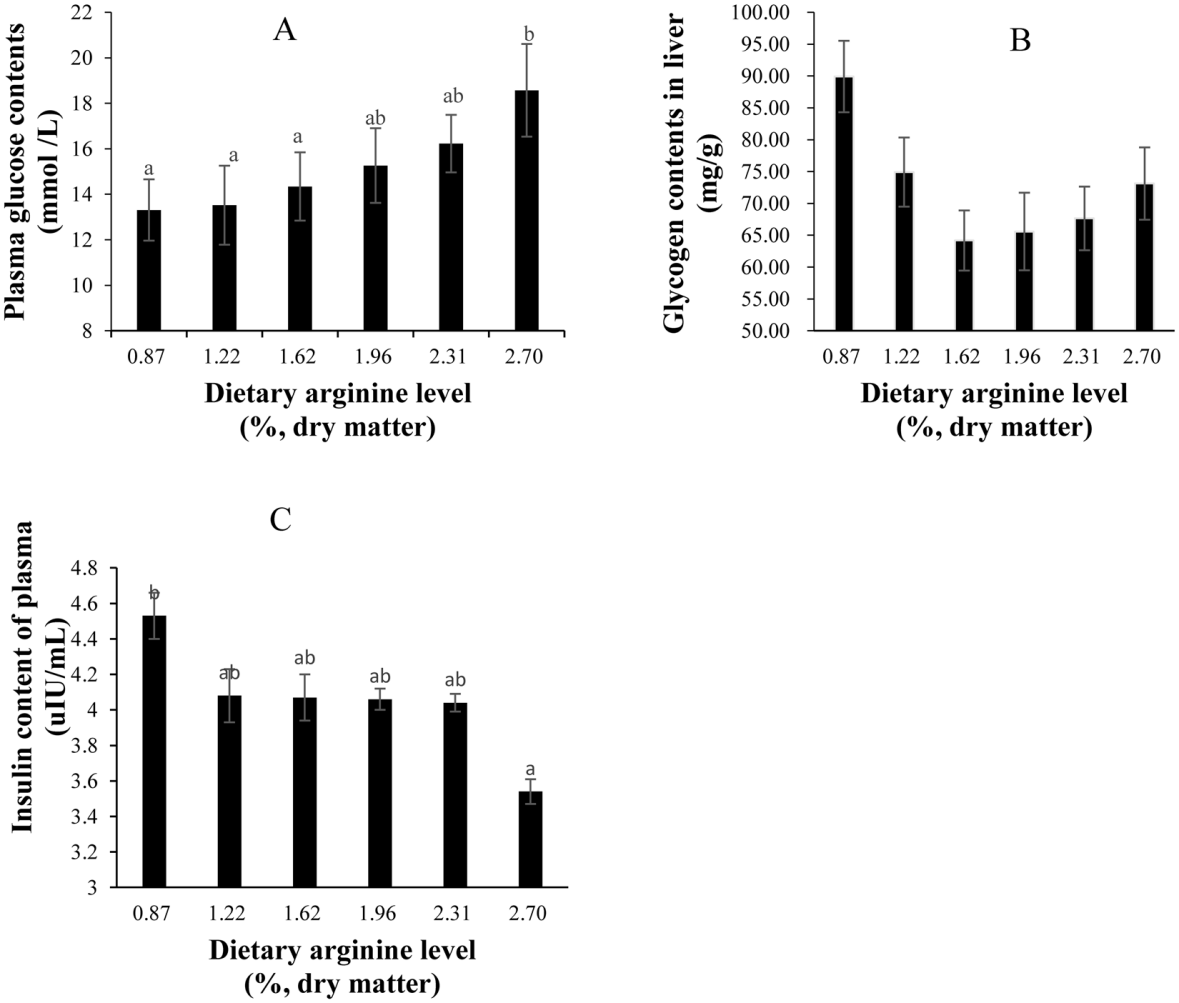
Plasma glucose (**A**) liver glycogen (**B**) and plasma insulin (**C**) contents of blunt snout bream fed diets with different arginine levels. Data are expressed as means with SEM. Value with different superscripts are significantly different (*P* < *0.05*).

The group fed with the optimal level of dietary arginine (1.62%) showed significantly higher relative expression of glucokinase (GK) compared with the expression observed in the control group (Fig. 2A). The relative expression of pyruvate kinase (PK) in the groups fed with dietary arginine levels of 1.96, 2.31 and 2.70% were significantly upregulated; the highest level of PK was observed in the 2.70% group (Fig. 2B). The relative expression of phosphoenolpyruvate carboxykinase (PEPCK) in the group fed with a dietary arginine level of 2.70% was significantly increased (Fig. 2C). The mRNA levels of glucose-6-phosphate (G6P) in the groups fed with dietary arginine levels of 1.62, 1.96, 2.31 and 2.70% were significantly upregulated; the highest level of G6P was found in fish fed with a dietary arginine level of 2.70% (Fig. 2D). Glycogen synthase (GS) expression showed a somewhat decreasing trend before showing an increasing trend; the lowest level of GS was observed in the group fed with a dietary arginine level of 1.62% (Fig. 2E). An excessive level of dietary arginine (2.70%) resulted in significantly lower glucose transporter 2 (GLUT 2) expression (Fig. 2F). An excessive level of dietary arginine (2.70%) resulted in significantly higher plasma glucose content and lower plasma insulin levels (Fig. 3A,C). Glycogen content was not significantly affected by the graded levels of dietary arginine (Fig. 3B).

### Lipid metabolism in the liver

**Figure 4:**
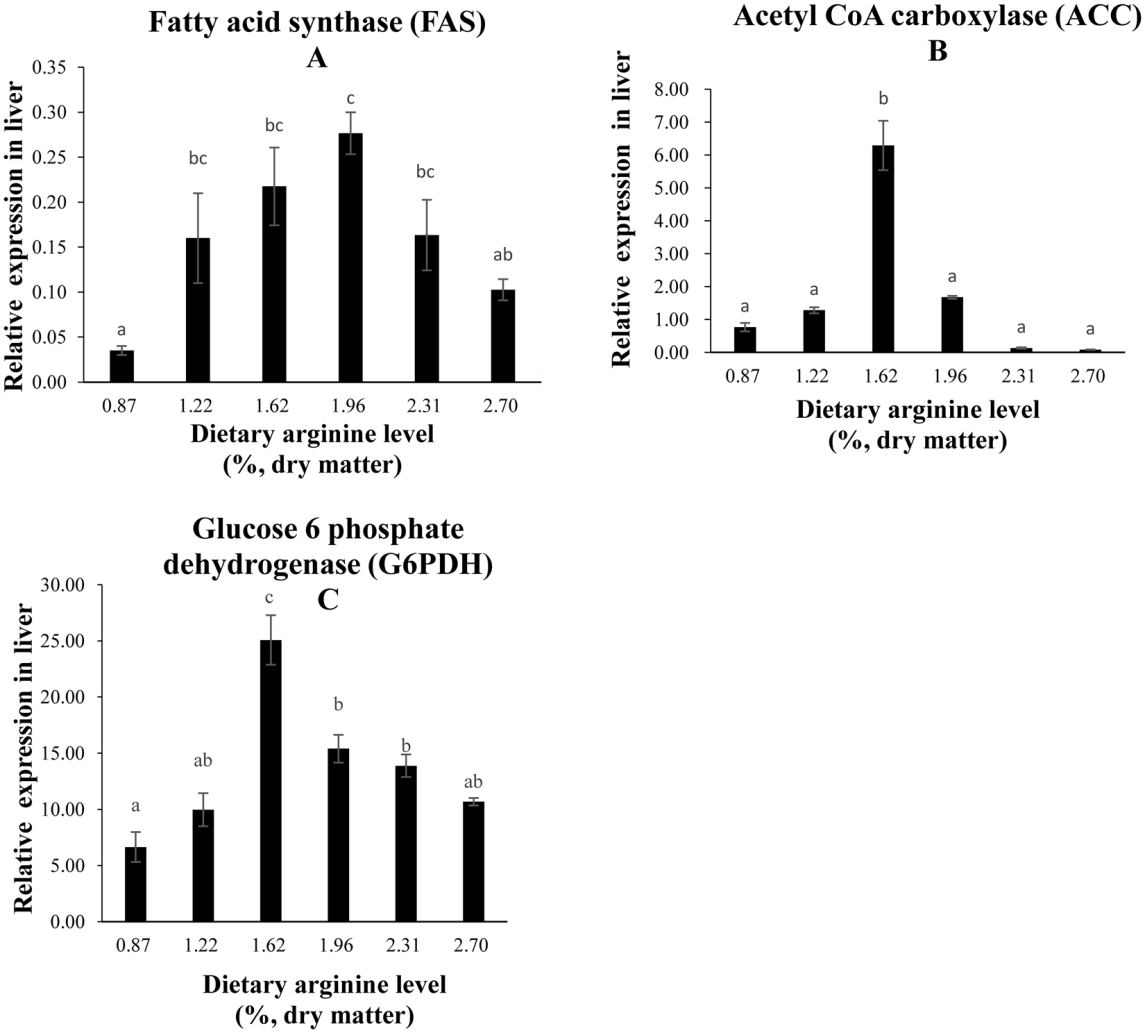
Relative expressions of lipid metabolism signaling pathway. Such as (**A**) FAS, (**B**) ACC, (**C**) G6PDH genes in the liver of blunt snout bream fed diets with different arginine levels. Data are expressed as means with SEM. Value with different superscripts are significantly different (*P* < *0.05)*.

**Figure 5:**
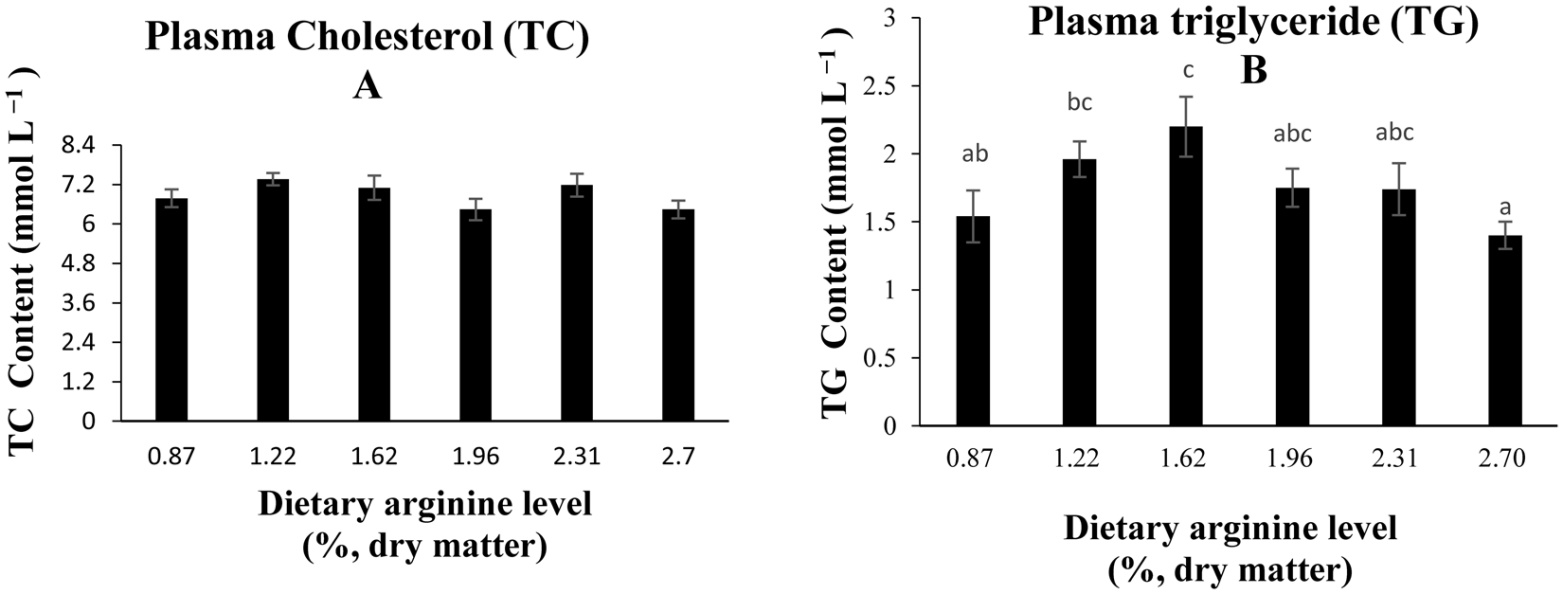
Plasma cholesterol (**A**) and triglyceride (**B**) contents of blunt snout bream fed diets with different arginine levels. Data are expressed as means with SEM. Value with different superscripts are significantly different (*P* < *0.05*).

The relative expression of fatty acid synthase (FAS) was significantly increased in the group fed with a dietary arginine level of 1.96% compared with that of the other groups (Fig. 4A). The optimal dietary arginine level (1.62%) significantly increased the levels of acetyl CoA carboxylase (ACC) and glucose-6-phosphate dehydrogenase (G6PDH) mRNA (Fig. 4B,C). Additionally, plasma triglyceride content (TG) was significantly increased in the groups fed with a dietary arginine level of 1.62% (Fig. 5B). Total cholesterol (TC) was not significantly affected by the graded levels of dietary arginine (Fig. 5A).

## Discussion

In our previous study, we reported that a high dietary arginine level (2.70%) led to the upregulation of S6K1 mRNA in the liver of blunt snout bream^2^. S6K1 is a serine kinase downstream of IRS-1, PI3K and Akt in the insulin signaling pathway, which is involved in the negative feedback regulation of insulin action^20^. In the obese state, high circulating concentrations of nutrients and cytokines promote target of rapamycin (TOR) activity in adipose tissue, which inhibits insulin signaling and causes insulin resistance through different mechanisms, including S6K1 kinase signaling^20^. IRS proteins act as messenger molecule-activated receptors of signaling pathways by interacting with Src homology 2 domains, which is an important step in insulin action^21^. In this study, we found that the relative expression of IRS-1 in the groups fed with dietary arginine levels of 1.96 and 2.31% was significantly upregulated compared with that of the control group. Similar results were reported in mice, that is, S6K1 mRNA was elevated, thereby inhibiting the expression of IRS-1 by inducing IRS-1 degradation^21^. PI3K is a downstream gene of IRS-1 and is also a key component of the insulin signaling pathway, which plays a central role in the metabolic actions of insulin^22,23^. In the present study, a high dietary arginine level (2.70%) downregulated the relative expression of PI3K compared to a lower (2.31%) dietary arginine level. Dysfunctional PI3K signaling would be expected to have deleterious effects on glucose homeostasis^22,23^. Impaired insulin-stimulated PI3K activity was observed in type II diabetes patients and in cultured fibroblast cells that were subjected to severe insulin resistance^22,23^. In addition, diabetic mice and insulin-resistant Zucker rats showed reduced PI3K activity^24^ or selective loss of PI3K signaling, which supports the role of PI3K in insulin resistance^25^. Akt (also known as protein kinase B), is particularly important in mediating several metabolic actions of insulin^26^. In this study, a high dietary arginine level (2.70%) significantly decreased the relative expression of Akt. Similar to this study^26–31^ decreased Akt kinase activity induced insulin resistance in rat skeletal muscle^27,28^. Akt has been shown to function in the insulin signaling cascade^26,29^. Some studies indicated that Akt can mediate insulin-stimulated glucose transport by binding to GLUT4-containing vesicles, thus decreasing GLUT4 translocation and causing insulin resistance^30,31^. In contrast, the total Akt protein levels were unchanged in muscle from type II diabetic patients^27^. Therefore, whether impaired Akt protein expression contributes to insulin resistance is still unknown. In the present study, a high dietary arginine level (2.70%) inhibited the relative expression of IRS-1, PI3K and Akt, which indicates that high dietary arginine levels result in a negative feedback mechanism that leads to insulin resistance in the liver of juvenile blunt snout bream. Nevertheless, physiological levels of L-Arg induce insulin production and improve insulin sensitivity in mammals, including humans^12,13,16^. This mechanism seems to be different from that found in mammals at the molecular level. Nevertheless, in mammals including human, L-Arg also induces insulin and improve insulin sensitivity, which promote the utilization of glucose^12,13,16^. Different from mammals and human, in our study, excess arginine level could decrease plasma insulin contents, and improve plasma glucose content. The mechanism seems to be different to the mammals at least at the molecular and insulin level. However, the literature is limited regarding fish, so the underlying mechanisms in fish are still not clear and further investigation is necessary.

GLUT 2 mediates bidirectional transport of glucose into hepatocytes, depending on hormonal and metabolic conditions^32,33^; this transport is the first step of glucose metabolism^32,34^. In our study, an excessive dietary arginine level (2.70%) resulted in significantly lower expression of GLUT2. Indeed, a decrease in GLUT2 levels can affect the capacity of glucose transfer between liver and blood, thereby affecting glucose metabolism in the liver^35^. Glycolysis is important in the glycometabolism pathway, and GK and PK are two important rate-limiting enzymes in this pathway^36–38^. In this study, an optimal dietary arginine level (1.62%) significantly upregulated the relative expression of GK. However, a high dietary arginine level (2.70%) significantly decreased the expression of GK mRNA. Our results indicate that optimal dietary arginine levels may improve hepatic glucose utilization in juvenile blunt snout bream, similar to their effects in mammals, but high dietary arginine levels may have a negative impact on hepatic glucose utilization, which could result in insulin resistance through a negative feedback mechanism. Previous research has shown that GK deficiency is linked to a form of diabetes in young men^39^. PK is the final step of glycolysis. In this study, the relative expression of PK in the groups fed with dietary arginine levels of 1.96, 2.31 and 2.70% was significantly upregulated; the highest level of PK was observed in the group fed with a dietary arginine level of 2.70%. These results suggest that in the omnivorous blunt snout bream fed with an optimal level of dietary arginine (1.62%), the liver is capable of strongly regulating the utilization of glucose but is not capable of synthesizing pyruvate. Furthermore, expression of GS and hepatic glycogen content in the liver was not significantly affected by dietary arginine levels, which indicates that synthetic glucose-6-phosphate was not used for the synthesis of hepatic glycogen. The present study suggests that glucose-6-phosphate is most likely related to the pentose phosphate pathway in this species of fish. In our study, high arginine supplementation upregulated the relative expression of PK in juvenile blunt snout bream. In contrast, Delwing *et al*. (2009) reported that acute administration of arginine inhibited PK activity in the cerebrum of rats^40^. This discrepancy could be due to species differences. Furthermore, Delwing’s study examined an acute administration, but our study employed a chronic or long feeding schedule, which may affect regulatory mechanisms^40^. To the best of our knowledge, this is the first study using fish to examine the impact of arginine on pyruvate kinase regulatory mechanisms; future work should examine the underlying mechanisms by which arginine affects PK.

The rate of gluconeogenesis is principally controlled by the activities of certain unidirectional enzymes, such as PEPCK and G6P^41^. In the present study, dietary arginine levels ranging from 1.22–2.31% did not significantly affect the relative expression of PEPCK. However, a high dietary arginine level (2.70%) significantly increased the mRNA expression of PEPCK. The results showed that a high dietary arginine level (2.70%) could accelerate the conversion of glucose-6-phosphate into pyruvate. G6P catalyzes the final step of gluconeogenesis, which is the production of free glucose from glucose-6-phosphate^41^. G6P mRNA levels were positively associated with dietary arginine levels in this study. Likewise, similar trends were observed in plasma glucose content; the highest level of plasma glucose content was observed in fish fed with high levels of dietary arginine (2.70%), implying that high dietary arginine levels could significantly elevate plasma glucose content compared with the control group (0.87%). Michael *et al*. (2000) reported that mice with a liver-specific insulin receptor knockout showed an increase in hepatic glucose production with high G6P and PEPCK expression levels^41^. In addition, the liver can produce glucose by breaking down glycogen through glycogenolysis and by synthesizing glucose from gluconeogenesis *de novo*^42^. In this study, hepatic glycogen content and expression of GS in the liver were not significantly affected by dietary arginine levels. Our results indicate that dietary arginine levels did not affect the glycogenolysis pathway. Therefore, the increase in plasma glucose content was caused by gluconeogenesis, not by glycogenolysis. In other words, a high dietary arginine level (2.70%) could upregulate the relative gene expression of PEPCK and G6P, which could enhance gluconeogenesis and result in high plasma glucose content in juvenile blunt snout bream.

An analysis of the insulin signaling pathway, including glycolysis and gluconeogenesis, showed that a high dietary arginine level (2.70%) resulted in the upregulation of S6K1 and inhibition of IRS-1, PI3K and Akt mRNA levels, which caused insulin resistance and elevated plasma glucose content. In another study, activation of the S6K1 pathway by nutrients or by prolonged insulin treatment led to insulin resistance due to increased IRS-1 serine phosphorylation and led to a reduction in IRS-1 function, as well as impaired activation of the PI3K/Akt pathway, thereby creating a negative feedback loop on insulin action in adipose tissue^43^. Interestingly, regarding the insulin signaling pathway and glucose metabolism, we found that a high dietary arginine level (2.70%) resulted in high expression of PEPCK in gluconeogenesis. However, high dietary arginine levels also resulted in high expression of PK in glycolysis. The results suggest a potential mechanism might be occurring in the “futile cycle” between PK and PEPCK. Some of the glucose-6-phosphate and pyruvate molecules have not been used as an energy source and, as a result, change each other persistently. Seiliez *et al*. (2011) also found a similar phenomenon in rainbow trout (*Oncorhynchus mykiss*)^44^; however, they observed a relationship between GK and G6P.

Insulin also promotes hepatic lipogenesis through several mechanisms and research has shown that arginine can affect lipid metabolism^10,13,45^. In our previous study, arginine supplementation (1.62% and 1.96%) increased whole body fat accretion in juvenile blunt snout bream^2^. G6PDH is the regulatory enzyme of the pentose phosphate pathway, which generated NADPH and is used in reductive biosynthetic processes such as the synthesis of fatty acids and steroids^46^. In this study, an optimal dietary arginine level (1.62%) significantly upregulated the relative expression of G6PDH in juvenile blunt snout bream. These results indicate that optimal dietary arginine levels heightened the pentose phosphate pathway, which showed that glucose-6-phosphate is mainly used in the pentose phosphate pathway to generate NADPH. This pathway result supports our hypothesis concerning the direction of glucose-6-phosphate in the glycolysis pathway. FAS and ACC have been shown to be critical to lipogenesis^47^. The relative expression of ACC and FAS was significantly upregulated in the groups fed with dietary arginine levels of 1.96 and 1.62%, respectively. Furthermore, TG content was also significantly increased in the group fed with a dietary arginine level of 1.62%. In another study, arginine supplementation (7.20% of dietary protein) resulted in the highest fat gain in Atlantic salmon (*Salmo salar*)^45^, which supports our current experimental results. Our study and Andersen’s study differ from studies carried out with mammals, which demonstrate a loss in fat mass after arginine supplementation^10–13^. To the best of our knowledge, most of the reports concerning the effect of arginine on fat gain in mammals have been based on observations of adult animals. However, in our study, the effect of arginine on fat gain has been based on juvenile animals. With this in mind, it will be interesting to study arginine supplementation in growing/adult blunt snout bream to assess whether arginine would have a whole body lipid-reducing effect.

**Figure 6:**
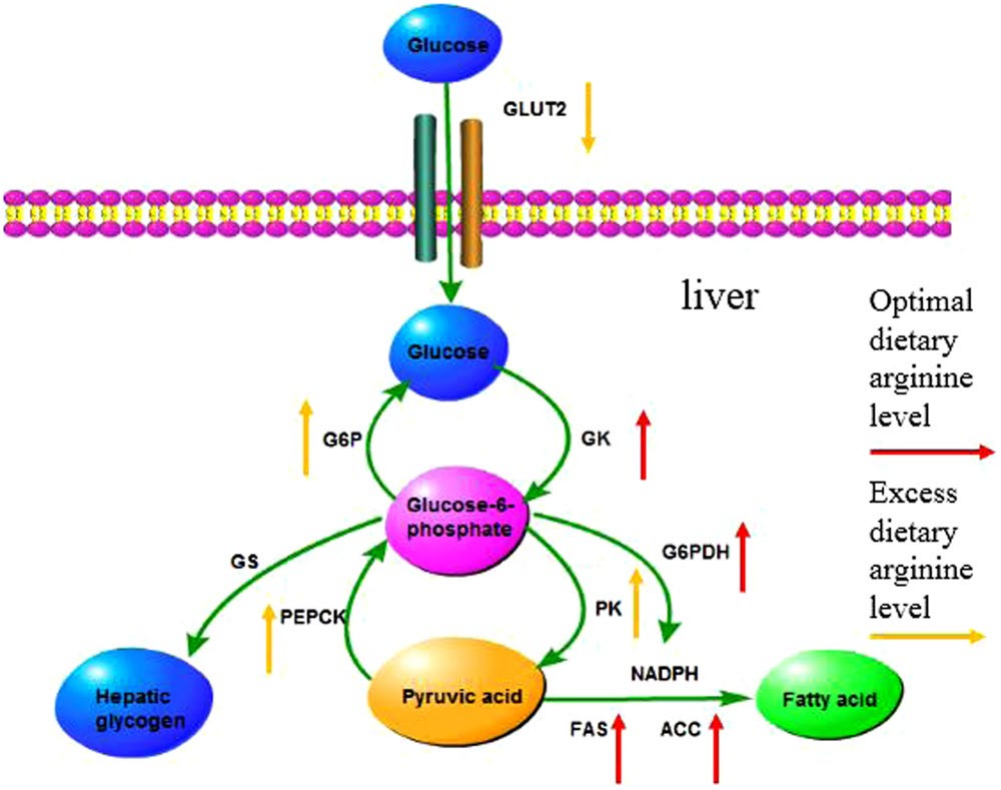
Glucose and lipid metabolism signaling pathway. Optimal dietary arginine level (1.62%) elevated the relative expression of Glucokinase (GK); Glucose-6-phosphate dehydrogenase (G6PDH); Fatty acid synthase (FAS); Acetyl CoA carboxylase (ACC). High dietary arginine level (2.70%) increased the relative gene expressions of Phosphoenolpyruvate carboxykinase (PEPCK); Glucose 6-phosphatase (G6P) and Pyruvate kinase (PK); lowered Glucose transporter 2 (GLUT 2).

**Figure 7:**
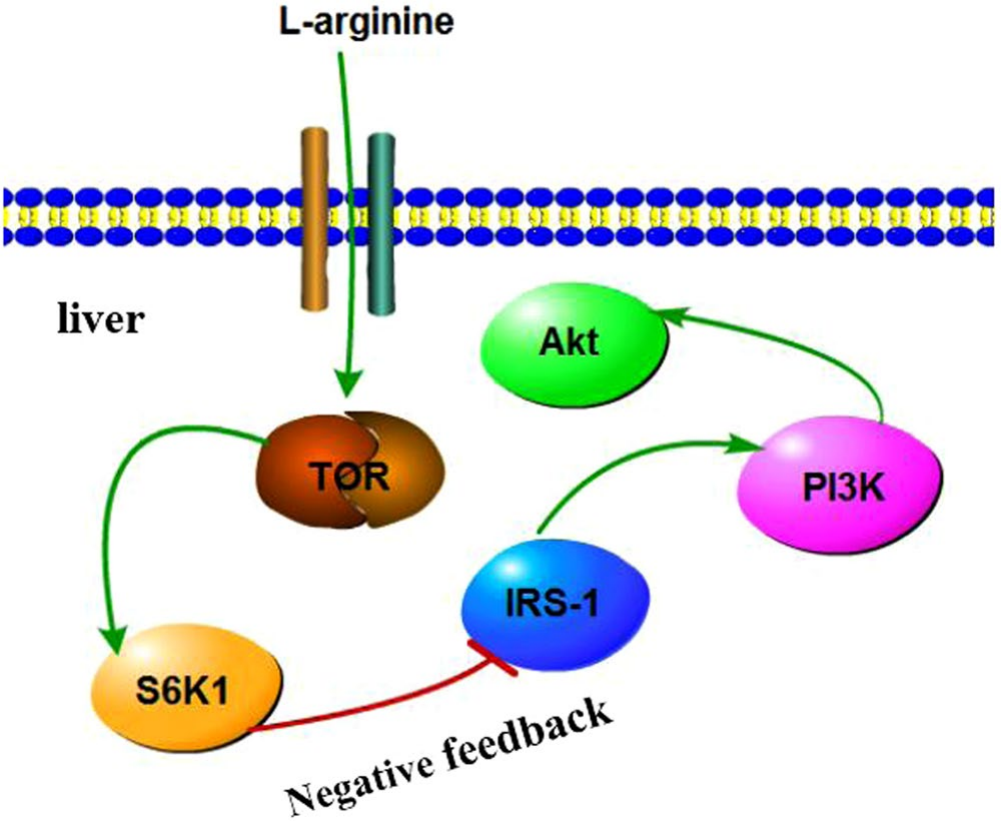
Insulin signaling pathway. Excess dietary arginine level (2.70%) resulted in protein S6 kinase 1 (S6K1) was over-expressed, which led to negative feedback in insulin resistance including inhibition of Insulin receptor substrate 1 (IRS-1); Phosphoinositide 3-kinase (PI3K) and Protein kinase B (Akt).

The present study demonstrated that chronic dietary arginine could affect the expression of genes involved in the insulin signaling pathway and lipid metabolism in juvenile blunt snout bream livers. In summary, an optimal dietary arginine level (1.62%) significantly elevated the relative expression of GK, converting intracellular glucose to glucose-6-phosphate, glucose-6-phosphate which was then mainly used for synthesis of fat, resulting in high whole body fat accretion and serum TG content; however, glucose-6-phosphatewas not used to synthesize pyruvate or hepatic glycogen (Fig. 6). A high dietary arginine level (2.70%) resulted in high plasma glucose, which may have contributed to a negative feedback mechanism that led to insulin resistance through the inhibition of IRS-1 and the upregulation of gluconeogenesis-related gene expression (Fig. 7). Furthermore, high dietary arginine levels could also result in the “futile cycle” between PEPCK and PK, but the mechanisms of the futile cycle in fish are still not clear and need further investigation.

## Materials and Methods

### Diet preparation

Six isonitrogenous and isoenergetic practical diets (33.0% crude protein, 7.0% crude lipid) were formulated to contain graded arginine levels using fish meal, rapeseed meal and corn gluten as protein sources and lecithin as a lipid source (Table 1); the arginine levels were at 0.87% (control), 1.22%, 1.62%, 1.96%, 2.31% and 2.70%. An amino acid profile of the experimental diets was formulated to simulate the whole body amino acid pattern of blunt snout bream expected for arginine (L-arginine 99%; Shanghai Feeer Technology Development Co. Ltd. China). Dietary arginine was replaced with equal proportions of glycine (Table 1).

**Table 1:** Formulation and proximate composition of the experimental diets (% dry matter).

Ingredients	Diet number					
	1	2	3	4	5	6
Fish meal^a^	5.00	5.00	5.00	5.00	5.00	5.00
Rapeseed meal^a^	5.00	5.00	5.00	5.00	5.00	5.00
Corn starch	12.10	12.10	12.10	12.10	12.10	12.10
Corn gluten^a^	22.00	22.00	22.00	22.00	22.00	22.00
Soybean oil	3.00	3.00	3.00	3.00	3.00	3.00
Soybean lecithin	2.00	2.00	2.00	2.00	2.00	2.00
Amino acid mix^b^	9.24	9.24	9.24	9.24	9.24	9.24
Choline chloride	0.10	0.10	0.10	0.10	0.10	0.10
Wheat meal^a^	22.00	22.00	22.00	22.00	22.00	22.00
Vitamin and mineral premix^c^	1.50	1.50	1.50	1.50	1.50	1.50
Monocalcium phosphate	3.00	3.00	3.00	3.00	3.00	3.00
Vitamin C	0.05	0.05	0.05	0.05	0.05	0.05
Microcrystalline cellulose	10.00	10.00	10.00	10.00	10.00	10.00
Ethoxy quinoline	0.01	0.01	0.01	0.01	0.01	0.01
Glycine	2.00	1.60	1.20	0.80	0.40	0.00
L-arginine	0.00	0.40	0.80	1.20	1.60	2.00
Bentonite	3.00	3.00	3.00	3.00	3.00	3.00
***Proximate analysis (% Dry basis)***						
L-arginine	0.87	1.22	1.62	1.96	2.31	2.70
Crude protein	33.64	33.68	33.84	33.34	34.76	34.59
Crude lipid	7.73	7.62	7.61	7.27	7.28	7.23
Ash	5.01	5.02	5.05	5.01	5.1	5.08

^a^Rapeseed meal obtained from Wuxi Tongwei feedstuffs Co., Ltd, Wuxi, China, crude protein 37.5%, crude lipid 1.4%; Corn gluten, obtained from Wuxi Tongwei feedstuffs Co., Ltd, Wuxi, China, crude protein 55.9%, crude lipid 3.3%; fish meal, obtained from Wuxi Tongwei feedstuffs Co., Ltd, Wuxi, China, crude protein 61.4%, crude lipid 9.3%; wheat meal obtained from Wuxi Tongwei feedstuffs Co., Ltd, Wuxi, China, crude protein 11.8%, crude lipid 1.2%.

^b^Amino acid premix (g/100 g diet): L-histidine, 0.22; L-isoleucine, 0.50; L-lysine, 1.57; L -phenylalanine, 0.2; L-threonine, 0.53; L-valine, 0.44; L-aspartic acid, 1.18; serine, 0.31; glycine, 1.55; alanine; 0.39; L -tyrosine,0.07; tryptophan, 0.12; glumatic acid, 0.14; proline 0.10. Amino acids obtained fromFeeer Co., LTD (Shanghai, China).

^c^Vitamin and mineral mix (IU or mg/ kgof diet): Vitamin A, 900 000 IU; Vitamin D, 250 000 IU; Vitamin E, 4500 mg; Vitamin K 3, 220 mg; VitaminB 1, 320 mg; Vitamin B 2, 1090 mg; Vitamin B 5, 2000 mg; Vitamin B 6, 5000 mg; Vitamin B 12, 116 mg; Pantothenate, 1000 mg; Folic acid,165 mg; Choline, 60 000 mg; Biotin, 50 mg; Niacin acid, 2500 mg;provided by Tongwei Feed Group Co. (Jiangsu, China).Supplied as L-form (99%, Shanghai Feeer Technology Development Co. Ltd., Shanghai, China).Mineral mix (g /kg of diet): calciumbiphosphate, 20 g; sodiumchloride, 2.6; potassium chloride, 5 g; magnesium sulphate, 2 g; ferrous sulphate, 0.9 g; zinc sulphate, 0.06 g;cupric sulphate, 0.02; manganese sulphate, 0.03 g; sodium selenate, 0.02 g; cobalt chloride, 0.05 g; potassium iodide, 0.004; and zeolite was used as a carrier.

### Experimental procedure

This study was approved by the Animal Care and Use Committee of Nanjing Agricultural University (Nanjing, China). All animal procedures were performed according to the Guideline for the Care and Use of Laboratory Animals in China. Juvenile blunt snout bream were obtained from the breeding farm of the Freshwater Fisheries Research Centre (FFRC) of the Chinese Academy of Fishery Sciences. Prior to the feeding trial, all fish were selected based on health and similarities in size and then cultured in floating net cages (1 m × 1 m × 1 m). The fish were fed a 33% protein and 7% lipid commercial diet (Wuxi Tongwei feedstuffs Co. Ltd., Wuxi China) for two weeks to allow the fish to adapt to the experimental feeds; fish were then fasted for 24 h, weighed, and then randomly distributed to 1 of 18 net cages with 20 fish in each cage for farm pond culture. Each experimental diet was randomly assigned to triplicate cages for 8 weeks. Fish were hand-fed to apparent satiation three times daily at 8:00, 12:00 and 16:00. During the experimental period, the water temperature ranged from 26 to 28 °C, pH values ranged from 7.3 to 7.8, dissolved oxygen concentrations ranged from 6.0 to 7.5 mg/L, ammonia nitrogen levels ranged from 0.005 to 0.009 mg/L and hydrogen sulfide levels ranged from 0.005 to 0.008 mg/L.

### Sample collection and chemical analysis

At the end of the feeding trial, fish were anesthetized using MS-222 (100 mg L^−1^; neutralized according to the method described by Tran-Duy^48^), weighed, and then blood samples were immediately collected from the caudal vein with disposable medical syringes. At the same time, liver samples were collected from the sampled fish. Plasma was separated by centrifugation (3500 × *g*, 10 min, 4 °C). Plasma and liver samples were stored at −80 °C until analysis.

Plasma glucose, TC and TG levels were determined using an automatic biochemical analyzer (Mindray BS-400, Mindray Medical International Ltd., Shenzhen, China) according to the glucose oxidase, cholesterol oxidase and GPO-POD methods, respectively. The glycogen content of the liver was determined using the liver glycogen assay kit (Jian Cheng Bioengineering Institute, Nanjing, China). Plasma insulin levels were determined using an automatic immunoassay analyzer (MAGLUMI 2000, Shenzhen snibe diagnostic Ltd, Shenzhen, china).

The relative expression of genes involved in the insulin signaling pathway and in lipid metabolism were determined using Real-time PCR analysis as described in our previous studies^49–51^. Briefly, total RNA extraction from the livers of juvenile blunt snout bream was performed by an RNAiso Plus kit (Takara, Dalian, China). After the quality and quantity of RNA were assessed using agarose gel electrophoresis at 1% and spectrophotometric analysis (A 260:280 nm ratio), complementary DNA (cDNA) was synthesized using a PrimeScript^TM^ RT reagent kit (Takara, Dalian, China). The transcript levels of IRS-1, PI3K, Akt, GK, GS, G6P, PEPCK, ACC, FAS, G6PDH, and GLUT2 were quantified using a 7500 Real Time PCR System (Applied Biosystems, USA).

Specific primers for key genes of the insulin signaling pathway and lipid metabolism were designed according to the partial cDNA sequences of these genes using the *M. amblycephala* transcriptome analysis (Table 2)^52^. β-actin was employed as a non-regulated reference gene, as it has previously been used in blunt snout bream studies^48,53^. All primers were synthesized by Shanghai Biocolor, BioScience &Technology Company, PR China. No changes in β-actin gene expression were observed in our investigation. The relative quantification of target gene expression was performed using Pfaffl’s mathematical model^54^.

**Table 2:** Primer sequences for qRT-PCR analysis.

Primer	Sequence Information	
	Forward primer (5′-3′)	Reverse primer (5′-3′)
β-actin	TCGTCCACCGCAAATGCTTCTA	CCGTCACCTTCACCGTTCCAGT
Akt^1^	GCTGGGTAAAGGCACGTTTG	CTCTCGGTGACCGTATGAGC
GS^2^	TTACACGGTCATTGCGTCCA	GACACAGCTCAGTCGGTGAA
PEPCK^3^	TCGCCTGGATGAAGTTCGAC	GTCTTGGTGGAGGTTCCTGG
IRS-1^4^	AACCTGGTTGGCATCTACCG	ATCAGCTGGAGCACGATAGC
G6PDH^5^	TGGAGAAACCTTTTGGCCGT	CTGGGTACCAAACGGCTCTT
GK^6^	GCTTCCACTGGGATTCACCT	CGACGTTATTGCCTTCAGCG
PK^7^	CGAGATTGAGAACGGAGGCA	GTCCTTCTCAGACACTGCGG
FAS^8^	GTTTGCCAACCGCTTGTCTT	GGCCATGGCGAATAGCATTG
ACC^9^	TAGCAGTGAGCATTGGCACA	CATCGCTGGCGTATGAGGAT
GLUT2^10^	CGGTGAAACCGAACAGGAGT	TTCTTTGAGATCGGGCCTGG
G6P^11^	TTCAGTGTCACGCTGTTCCT	TCTGGACTGACGCACCATTT
PI3K^12^	GGCGTAACATCCAGCTTTGC	GCTCCTGGAAGCTGGGTAAC

Note: ^1^Akt: Protein kinase B; ^2^GS: Glycogensynthase; ^3^PEPCK: Phosphoenol pyruvate carboxykinase; ^4^IRS-1: Insulin receptor substrate 1; ^5^G6PDH: Glucose-6-phosphate dehydrogenase; ^6^GK: Glucokinase; ^7^PK: Pyruvate kinase; ^8^FAS: Fatty acid synthase; ^9^ACC: Acetyl CoA carboxylase; ^10^GLUT2: Glucose transporter 2; ^11^G6P: Glucose 6-phosphatase; ^12^PI3K: Phosphoenolpyruvate carboxykinase.
