# Publisher Correction: Dietary arginine affects the insulin signaling pathway, glucose metabolism and lipogenesis in juvenile blunt snout bream *Megalobrama amblycephala*

**DOI:** 10.1038/s41598-018-20030-y

**Published:** 2018-02-02

**Authors:** Hualiang Liang, Habte-Michael Habte-Tsion, Xianping Ge, Mingchun Ren, Jun Xie, Linghong Miao, Qunlan Zhou, Yan Lin, Wenjing Pan

**Affiliations:** 1 Wuxi Fisheries College, Nanjing Agricultural University, Wuxi, 214081 China; 2 Key Laboratory for Genetic Breeding of Aquatic Animals and Aquaculture Biology, Freshwater Fisheries Research Center (FFRC), Chinese Academy of Fishery Sciences (CAFS), Wuxi, 214081 China; 3 Division of Aquaculture, College of Agriculture, Food Science and Sustainable Systems (CAFSSS), Kentucky State University, 103 Athletic Road, Frankfort, KY 40601 USA

Correction to: *Scientific Reports* 10.1038/s41598-017-06104-3, published online 11 August 2017

In this Article, Mingchun Ren was not correctly recognised as a corresponding author. For correspondence and requests for materials, please contact Xianping Ge (gexp@ffrc.cn) or Mingchun Ren (renmc@ffrc.cn).

This has been corrected in the PDF and HTML versions of the Article.

